# CpGPAP: CpG island predictor analysis platform

**DOI:** 10.1186/1471-2156-13-13

**Published:** 2012-03-02

**Authors:** Li-Yeh Chuang, Cheng-Huei Yang, Ming-Cheng Lin, Cheng-Hong Yang

**Affiliations:** 1 Department of Chemical Engineering & Institute of Biotechnology and Chemical Engineering, I-Shou University, Kaohsiung, 8004, Taiwan; 2 Department of Electronic Communication Engineering, National Kaohsiung Marine University, Kaohsiung, 81157, Taiwan; 3 Department of Electronic Engineering, National Kaohsiung University of Applied Sciences, Kaohsiung, 80778, Taiwan; 4 Department of Network Systems, Toko University, Chiayi, 61363, Taiwan

## Abstract

**Background:**

Genomic islands play an important role in medical, methylation and biological studies. To explore the region, we propose a CpG islands prediction analysis platform for genome sequence exploration (CpGPAP).

**Results:**

CpGPAP is a web-based application that provides a user-friendly interface for predicting CpG islands in genome sequences or in user input sequences. The prediction algorithms supported in CpGPAP include complementary particle swarm optimization (CPSO), a complementary genetic algorithm (CGA) and other methods (CpGPlot, CpGProD and CpGIS) found in the literature. The CpGPAP platform is easy to use and has three main features (1) selection of the prediction algorithm; (2) graphic visualization of results; and (3) application of related tools and dataset downloads. These features allow the user to easily view CpG island results and download the relevant island data. CpGPAP is freely available at http://bio.kuas.edu.tw/CpGPAP/.

**Conclusions:**

The platform's supported algorithms (CPSO and CGA) provide a higher sensitivity and a higher correlation coefficient when compared to CpGPlot, CpGProD, CpGIS, and CpGcluster over an entire chromosome.

## Background

CpG islands are stretches of typically unmethylated DNA with a high content of the two nucleic acids Cytosine (C) and Guanine (G), i.e., a high CG content relative to the bulk DNA. In typical mammalian genomes, many CpG islands (about 40%) are found in the promoter region. Of the estimated 45,000 CpG islands in the human genome, the overwhelming majority is found at the 5' end of genes. Identification and cloning of these CpG islands has proven very useful for finding and isolating genes. The CpG sites in the CpG islands of promoters are unmethylated if genes are expressed. It has thus been speculated that methylation of these sites plays a crucial role in gene expression [1].

CpG islands were originally identified by Tykocinski and Max as small areas that contain the restriction enzyme *HpaII *in the genome and were thus called *HpaII *Tiny Fragment (HTF) islands [2]. Gardiner-Garden and Frommer (GGF) defined GpG islands as a DNA sequence with a length exceeding 200 bp, a GC content in that region of greater than 50%, and an Observed/Expected (O/E) ratio of greater than 0.6 [3]. After Takai and Jones re-evaluated the three parameters in the GGF definition of CpG islands, they proposed a new set of criteria (GC content > 55%, O/E ratio > 0.65, length > 500 bp) [4]. This algorithm can effectively exclude false positives from short repetitive sequences (e.g., *Alu*) [5].

Recently, various tools and methods have been proposed to predict CpG islands, e.g., CpGPlot [6], CpGProD [7], CpGIS [8] and CpGcluster [9], and the epigenome prediction method [10]. Rice et al. proposed the CpGPlot program, which plots CpG-rich areas and reports all CpG-rich regions [6]; CpGPlot yields the lowest sensitivity values. This could indicate that CpGPlot does not predict any islands in the target sequence and does not achieve a good performance. Ponger et al. proposed CpGProD, an application for identifying mammalian promoter regions associated with CpG islands in large genomic sequences. CpGProD shows the predicted probability over a transcription start site (TSS) chart, and the mammalian promoter regions associated with CpG island information [7]. CpGProD uses a sliding window technique, where the size of the window greatly influences the results, thus making its prediction unreliable [11]. Takai and Jones proposed the CpGIS algorithms, which merges two CpG islands when they were less than 100 bp apart and the merged CpG island still mets the GGF criteria [8]. CpGIS provides a graphical map of the CpG dinucleotide distribution. Hackenberg et al. proposed CpGcluster, which uses only integers for calculations. It is thus a fast and efficient method for predicting significant clusters of CpG dinucleotides [9]. Bock et al. proposed an epigenome prediction method, which derives quantitative scores of "CpG island strength" that incorporate epigenetic and functional aspects to help resolve the problem that some current CpG island criteria incur significant disadvantages [10].

In this study, we present a web-based application called CpGPAP (CpG island predictor analysis platform) which uses the complementary particle swarm optimization (CPSO) [12] and a complementary genetic algorithm (CGA) [13] in combination with some methods from the literature. CpGPAP produces graphic visualizations of the GC%, the O/E ratio, the distribution of CpG and the probability of a CpG island overlapping with a transcription start site. Furthermore, the proposed method allows users to freely select parameter settings and easily view CpG island information. This predictor platform can be of assistance to biologists involved in the study of CpG islands.

### Implementation

The proposed CpGPAP interface was developed in JSP with Java script (jdk1.6.0_071.4.0). It is freely available at http://140.127.113.93/CpGPAP/.

## Results

### Input module

**Figure 1:**
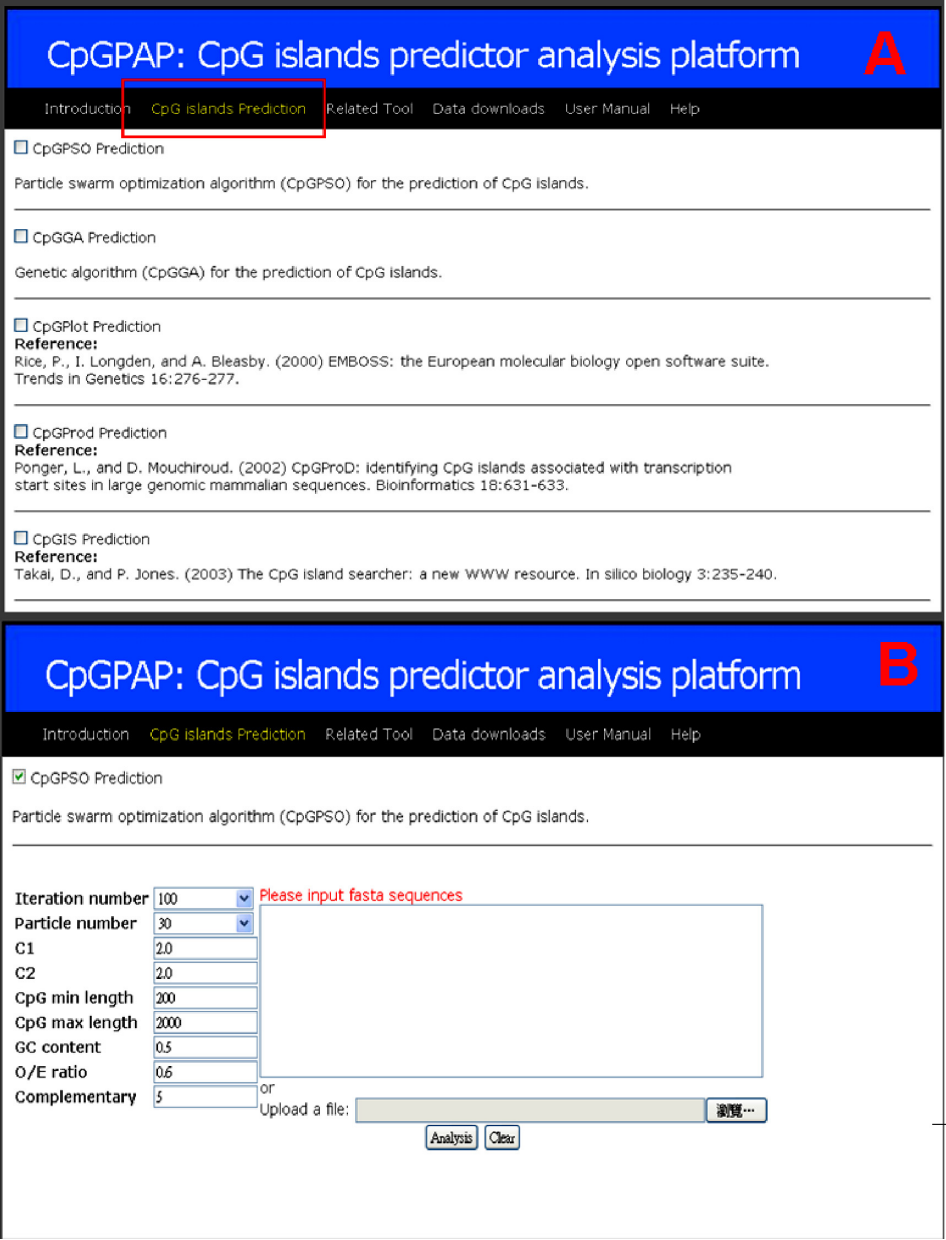
**CpGPAP platform flowchart**. **A: **Selection of the optimization algorithm for predicting CpG islands. **B: **Parameter settings for the optimization algorithm, CpG island related parameters and input sequence

The input module of the CpGPAP platform contains three main functions. First, the prediction algorithm, i.e., CPSO, CGA, CpGPlot, CpGProD or CpGIS is selected (Figure 1A). Then, the optimization algorithm's parameters are set, which include algorithm-related and CpG island-related parameters (CpG island length, GC content and O/E ratio). FASTA sequences with the four nucleotides adenine (A), thymine (T), cytosine (C) and guanine (G) are accepted as input sequences (Figure 1B).

### Analysis module

**Figure 2:**
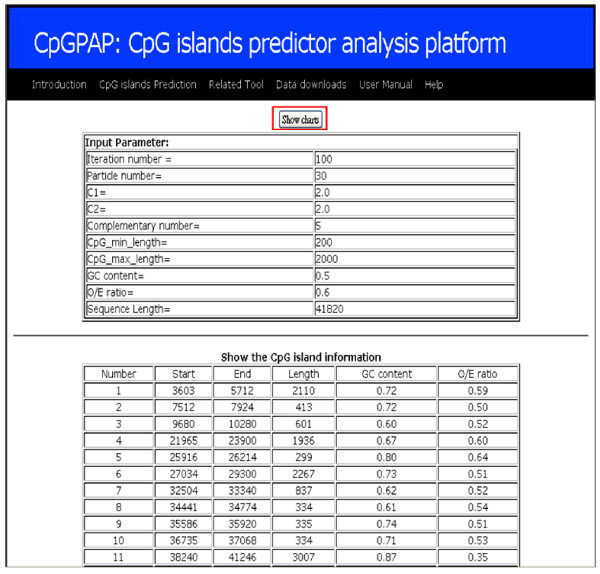
**Prediction results showing CpG island-related information**. such as number of CpG islands, CpG island length, start and end position, input parameters, O/E ratio, and GC content

Figure 2 shows the optimization algorithm parameter settings. Subsequently, the predicted CpG island results, including the number of CpG islands, the CpG island start and end positions, the length of the CpG island, the GC content and the O/E ratio, are shown. The "show chart" function can be used to further explore the results. The function visualizes the CpG island mapping, and depicts the CpG dinucleotides distribution, the GC content, the O/E ratio distribution, and the CpG island sequence distribution.

### Output module

**Figure 3:**
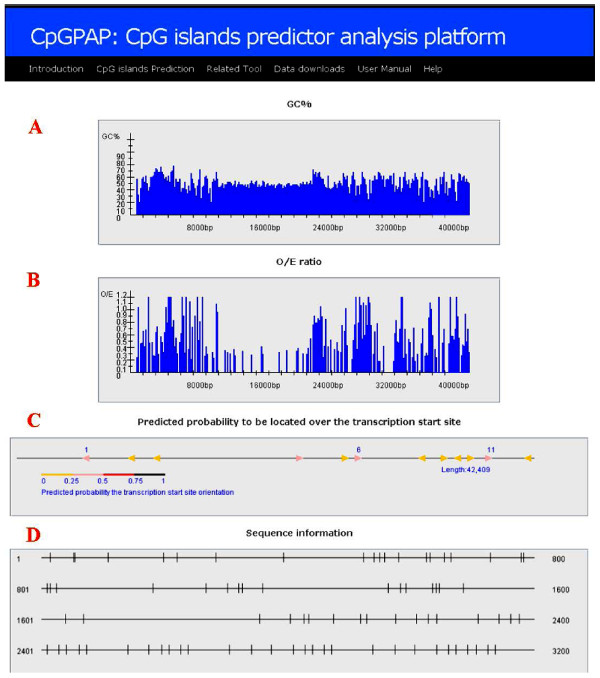
**Visualization of the CpG island prediction results**. **A: **GC% chart shows the GC content distribution in the input sequence. **B: **O/E ratio chart shows the O/E ratio distribution in the input sequence. **C: **TSS chart shows the probability of the predicted CpG island overlapping with a transcription start site. **D: **CpG chart shows the CpG nucleic and CpG island distribution

To clearly determine the distribution of CpG islands, the CpGPAP platform generates a graphic visualization once the CpG islands prediction results are complete. The design of the chart is mainly based on the GGF criteria; we thus focused on GC content (GC%), O/E ratio and CpG island length design. The prediction results can be divided into four main types of CpG island-related information. (1) GC% charts are calculated from the input sequence with a calculation processed every 50 bp on average; (2) O/E ratio charts are calculated through the same process as GC%; (3) the predicted probability of being over the transcription start site chart is obtained by providing the CpGProD (http://pbil.univ-lyon1.fr/software/cpgprod.html); and (4) the distribution of CpG charts shows the predicted CpG islands resulting in the predicted genome sequence position, including the CpG island overlap input sequence position, the number of CpG islands and all connections to the CpG nucleic position. All of the above results are shown in Figure 3. Theoretically, in the large-scale computational analysis of CpG islands, the CpGPAP platform can accept any sequence input and dataset size. However, to avoid data transfer errors, we limited the "show charts" function to display graphic sequence information of 50 kb or less. The graphic visualization allows researchers to set related parameters accurately and obtain better prediction results. A stand-alone version is also available for download with no input sequence size limitation.

### Related tools and dataset download

The CpGPAP platform also includes CpGPlot, CpGProD, CpGIS, and CpGcluster all of which are related CpG island prediction tools. We furthermore included dataset links for related downloads, including the National Center for Biotechnology Information (NCBI), the University of California Santa Cruz (UCSU), RegulonDB, the Database of Transcriptional Start Sites (DBTSS), and the European Bioinformatics Institute (EMBL-EBI) for the users convenience.

## Discussion

DNA methylation is a major epigenetic modification of the genome that affects basic biological functions [14] such as the gene expression [15] and human diseases, especially cancer [16,17]. DNA methylation occurs most commonly in a CpG sequence called CpG islands. Hence, we developed a visualization tool that depicts CpG island distribution charts to facilitate methylation related studies.

Several methods are already available to determine a CpG island region, e.g., CpGPlot, CpGProD, CpGIS, and CpGcluster. However, these methods, with the exception of CpGIS, do not simultaneously provide the GC content, the O/E ratio distribution and the CpG islands sequence distribution. A comparison of currently used platforms is shown in Table 1. The CpGPlot, CpGProD, CpGIS, and CpGcluster methods used to predict CpG islands are discussed in the methods section. These tools, with the exception of CpGcluster, use methods that are based on three parameters, namely the GC content, the O/E ratio and the CpG island length, to predict CpG islands. Most use the sliding window technique with the GC content, the O/E ratio and length thresholds as the main parameters, while CpGcluster uses the distance between CpG dinculeotides. The sliding window technique is similar to brute force searches and thus yields high *SP *values. Han et al. [18,19] and Hackenberg et al. [9,20] identified several disadvantages of methods based on the sliding window technique: (1) CpG islands identified by these methods generally do not start and end with a CpG dinucleotide [21]. (2) The number and length of the CpG islands is obtained based on the window size and the step size. If the window is large, several short and loosely distributed CpG islands may be merged into a larger one. (3) The run time for these methods is relatively long. The CpGcluster method predicts CpG islands based on the physical distance between CpG dinucleotides [20]. Although CpGcluster can identify some short CpG islands, a high number of false positives (*FP*) is obtained over the entire genome [18]. In addition, other problems exist for CpGCluster: (1) Search results are dependent on the composition of the sequence scanned, i.e., a CpG island identified in one sequence may be discarded when planted into another sequence with a different composition. (2) It also has a low prediction sensitivity [9]. This shows that there is still room for improvement.

**Table 1:** Comparison of different software functions for CpG island

Year of publication		2011	2006	2003	2002	2000
**Software**		**CpGPAP**	**CpGcluster**	**CpGIS**	**CpGProD**	**CpGPlot**

**Reference**		[22]	[8]	[7]	[6]	[5]

**Availability**		http://140.127.113. 93/CpGPAP/	http://bioinfo2.ugr. es/CpGcluster/	http://cpgislands.usc.edu/	http://pbil.univ-lyon1.fr/softwar e/cpgprod_quer	http://www.ebi.ac.uk/Tools/emboss/cpgp

**F** **u** **n** **c** **t** **i** **o** **n**	Type	Web-based	Web-based	Web-based	Web- based	Web- based
	
	Parameters settings	√	√	√	√	√
	
	CpG island result	√	√	√	√	√
	
	CpG dinucleotides	√		√		
	
	TSS	√			√	
	
	O/E ratio bar	√				√
	
	GC% bar	√				
	
	Upload sequence	√			√	√
	
	Data integrator	√				
	
	Standalone	√				
	
	version	√	√	√	√	
	
	Method	CPSO and CGA	cluster	Sliding window	Sliding window	Sliding window

TSS: transcription start site. CPSO: complementary particle swarm optimization. CGA: complementary genetic algorithm

In order to validate the performance of the complementary PSO (CPSO) and complementary GA (CGA) (see Additional file 1: Algorithm CGA), i.e., the two algorithms supported by our platform; we compared them to other methods on the human chromosomes 21 and 22. Chromosomes 21 and 22 are widely used in the literature, so we used the available data on the chromosomes for the validation process. Calculations for all the other human chromosomes can be found in [22]. Table 2 shows a comparison to the other methods. Information includes the chromosome length, the total length of the CpG islands, the number of islands predicted, the coverage (%), the island length (average, minimum, and maximum), the GC content, and CpG island O/E ratio values.

**Table 2:** Comparison of the number of CpG islands identified in the human genome with different methods *(NCBI.36)*

				*Chromosome 21*				
	
	*CpGPlot*	*CpGcluster*	*CpGProD*	*CpGIS*	*PSO*	*CPSO*	*GA*	*CGA*
Chromosome Length (bp)				46,944,329				

Total length of CpG islands	347,334	639,161	1,072,192	1,280,505	1,564,596	1,607,472	1,262,449	1,589,629

Number of islands predicted	973	2,703	1,091	3,704	2,648	2,813	2,513	3,304

Island coverage (%)	0.73	1.36	2.28	2.73	3.3	3.4	2.68	3.39

Island length (bp)								

Average	357	237	983	346	591	571	502	482
	
Minimum	101	8	500	200	202	202	201	201
	
Maximum	3,047	3,028	6,732	1,948	4,020	4,035	6,126	10,687
	
GC-content ± SD (%)	62.17 ± 0.07	65.49 ± 0.07	54.49 ± 0.06	57.98 ± 0.04	53.73 ± 0.05	53.72 ± 0.05	54.24 ± 0.05	55.07 ± 0.05

CpG island O/E ratio ± SD	0.84 ± 0.1	0.87 ± 0.3	0.63 ± 0.1	0.68 ± 0.1	0.64 ± 0.08	0.65 ± 0.08	0.68 ± 0.1	0.71 ± 0.1

				***Chromosome 22***				
	
	***CpGPlot***	***CpGcluster***	***CpGProD***	***CpGIS***	***PSO***	***CPSO***	***GA***	***CGA***

Chromosome Length (bp)				49,691,432				

Total length of CpG islands	679,803	522,748	2,067,653	2,842,255	2,802,675	2,907,983	2,251,454	3,085,715

Number of islands predicted	1,642	2,186	1,903	6,875	4,571	4,882	3,902	4,985

Island coverage (%)	1.36	1.05	4.16	5.71	5.64	5.85	4.53	6.20

Island length (bp)								

Average	414	239	1,087	413	613	596	577	619

Minimum	200	8	500	200	198	202	201	201
	
Maximum	7,902	7,774	8,363	3,339	4,076	4,076	5,340	5,905
	
GC-content ± SD (%)	63.70 ± 0.08	70.23 ± 0.08	55.84 ± 0.07	55.12 ± 0.06	54.50 ± 0.07	54.46 ± 0.07	55.21 ± 0.05	56.15 ± 0.06
	
CpG island O/E ratio ± SD	0.84 ± 0.1	0.95 ± 0.3	0.62 ± 0.1	0.68 ± 0.1	0.63 ± 0.05	0.63 ± 0.05	0.64 ± 0.1	0.68 ± 0.1

SD: Standard Deviation. Proportion (%) of the chromosome sequence covered by methods

Table 2 indicates that the number of CpG islands predicted by CpGIS is the highest for chromosomes 21 (3,704) and 22 (6,875). However, the total number of CpG islands does not represent a better prediction ability of this method since the average length of CpG islands predicted by CpGIS (346 bp and 413 bp for chromosome 21 and 22, respectively) is shorter than in our supported algorithms CPSO obtained a result of 571 bp and 596 bp for chromosomes 21 and 22, respectively. CGA obtained 482 bp and 619 bp for chromosomes 21 and 22, respectively. This indicates that the total length of the CpG islands predicted in chromosomes 21 and 22 by CpGIS is shorter than the length predicted by CPSO and CGA. In addition, when compared to the methods from the literature, the islands predicted by CPSO covered a larger region (3.4% and 5.85%) in chromosomes 21 and 22, respectively. The percentages of the region covered by CGA are 3.39% and 6.20% in chromosomes 21 and 22, respectively. The supported algorithms' average values for the GC content, the O/E ratio and the length of the predicted CpG islands all conform to the GGF criteria.

In general, around 80% of all CpG dinucleotides are methylated in mammalian genomes [23]. The lack of methylation is thus a very good indicator of the function of a CpG island [19,22]. The analysis platform was used to investigate the CpG island distribution in a DNA genome and obtained better prediction results for CpG islands than the other methods it was compared to. The accuracies (*ACC) *of the supported algorithms were also higher [24].

### Advantages of CpGPAP

CpGPAP has several advantages over other analysis platforms. Firstly, the platform-supported algorithms reach a high correlation coefficient (*CC*) (See Additional file 1: Table S1, Table S2 and Table S3). Secondly, CpGPAP provides an easily accessible interface for convenient visualization and analysis of genomic CpG islands. Furthermore, CpGPAP integrates currently existing tools and provides datasets for users to download. The supported algorithms (CPSO and CGA) predict CpG islands faster than other methods. The platform also provides a graphical overview of the putative islands. A stand-alone version with no limitations on the input sequence length is also available. This stand-alone version includes a visual display function. CpGPAP is thus a very useful tool for the detection and analysis of genomic CpG islands.

## Conclusions

We propose a novel CpG island prediction platform, CpGPAP, in which the platform's supported algorithms (CPSO and CGA) provide a higher sensitivity (*SN*) and a correlation coefficient (*CC*) as compared to the CpGPlot, CpGProD, CpGIS and CpGcluster platforms over an entire chromosome. CpGPAP integrates relevant approaches from the literature to provide the user with more options. The incorporated algorithms (CPSO and CGA) have a lower computational complexity than the other platforms in the literature. We integrated the three related prediction tools CpGPlot, CpGProD and CpGIS and provided dataset links for easy access. The CpG island prediction parameters can be selected freely. We believe that the proposed predictor platform can be of assistance to biologists involved in the study of CpG islands.

## Methods

### Supported algorithms

To predict CpG islands, the CpGPAP analysis platform includes two separate algorithms, CPSO and CGA. It also incorporates internet links to other prediction methods, namely CpGIS, CpGPlot and CpGProD. The development of the analysis platform is a continuation of our previous CpG island study [24].

PSO is a population-based stochastic optimization algorithm, which was developed by simulating the social behavior of organisms [12]. In PSO, each particle in the search space can be considered to be an individual bird in a flock, which changes its position based on its memory and its knowledge of its neighbors. Each particle from a swarm represents a candidate solution. The individual best value (*pbest_i_*) is the position of the *i*-th particle with the highest fitness at a given iteration; the best position of all *pbest *is called *gbest*. Particles use their individual memory (*pbest*) and the swarm's knowledge (*gbest*) as a whole to move around a multidimensional search space until the termination condition is reached. PSO has been successfully applied in many fields, including operon [25] and CpG island prediction [24], amongst others. The pseudo-code of PSO for the prediction CpG island is shown in Additional file 1: Figure S1.

GA is a stochastic search algorithm modeled after the process of natural selection that underlies biological evolution [13]. The standard GA procedure applies the following genetic operators: chromosome encoding and initialization, selection, crossover and mutation, which is the process by which a whole generation of new offsprings is computed. By applying genetic operators on strings in the mating pool, a new population of strings is formed in the next generation. The implementation of the genetic operators is repeated in each subsequent generation until a termination condition is reached. GAs have been successfully applied in many fields, e.g. microarray data exploration [26] and SNP interaction studies [27]. The pseudo-code of the GA for CpG island prediction is shown in Additional file 1: Figure S2.

If the PSO and GA search processes fall into a local optimum for five consecutive generations, the complementary concept is used to leave this local region and re-enter the global search. Additional file 1: Figure S3 shows the flowchart of the complementary GA (CGA), and Additional file 1: Figure S4 shows the flowchart of the complementary PSO (CPSO) as well as an illustrative example of how the CGA algorithm works. In addition, we integrated three sliding windows methods (CpGIS, CpGPlot and CpGProD) in the CpGPAP platform to predict CpG islands. These methods use the GC content, the O/E ratio and CpG island length as the main parameters to predict CpG islands.

### System overview

**Figure 4:**
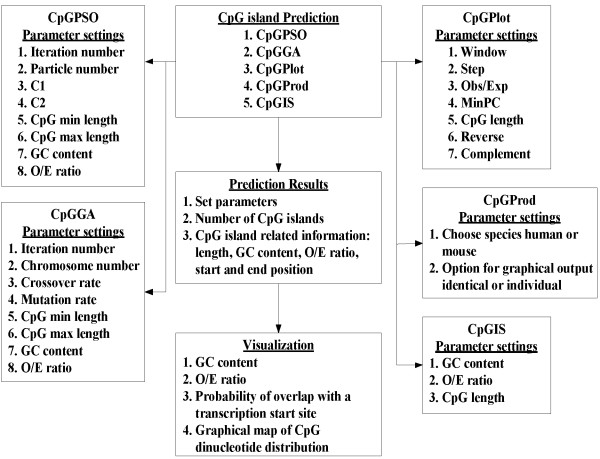
**CpGPAP system flowchart**.

Using the CpGPAP platform involves three steps. First, users select the optimization algorithms used to predict the CpG islands (Figure 1A). Then, the optimization algorithm's parameters and CpG island related parameters are set and the input sequence is uploaded (Figure 1B). In a final step, CpG island-related information predicted by the algorithm, such as length, start and end position, input parameters, O/E ratio, GC content, etc., is display (Figure 2). Users can choose whether to display a visualization of the prediction results (Figure 3), and CpGPAP parameters can be freely modified. Figure 4 shows the structure and flowchart of the CpGPAP system. In addition, while the other algorithms were initially designed based on the GGF criteria (i.e., GC content ≥ 50%, O/E ratio ≥ 0.6, and CpG island length > 200 bp), the parameters related to CpG islands can be freely modified in CpGPAP.

### Availability and requirements

Project home page: http://140.127.113.93/CpGPAP/

**Operating system(s): **Operating systems with web browser.

**Programming language: **Java, javascript, CSS and HTML.

**Other requirements: **Java 1.5.0 (or later).

**License: **none for academic users. For any restrictions regarding the use by non-academics please contact the corresponding author.

## Authors' contributions

LYC conceived of the study and drafted the manuscript. CHY participated in the design and helped to draft the manuscript. MCL participated in the design of the study and performed the analysis. CHY participated in its design and coordination and helped to draft the manuscript. All authors read and approved the final manuscript.

## Supplementary Material

Additional file 1 **Table S1**. Comparison of different methods for CpG island prediction (CPSO). **Table S2**. Comparison of different methods for CpG island prediction (CpGGA). **Table S3**. Comparison of different methods on the number of CpG islands identified in the entire human genomes. **Supplementary Methods**. Algorithm CGA. **Figure S1**. The pseudo-code for the PSO. **Figure S2**. The pseudo-code for the GA. **Figure S3**. Flowchart for the complementary GA. **Figure S4**. Flowchart for the complementary PSO [3,13,28,29]. Click here for file
