# Supplementary material for: CpGPAP: CpG island predictor analysis platform
Source: BMC Genet. 2012 Mar 2;13:13. doi: 10.1186/1471-2156-13-13 (PMC3313849; doi:10.1186/1471-2156-13-13)
Supplement: Additional file 1 — Table S1. Comparison of different methods for CpG island prediction (CPSO). Table S2. Comparison of different methods for CpG island prediction (CpGGA). Table S3. Comparison of different methods on the number of CpG islands identified in the entire human genomes. Supplementary Methods. Algorithm CGA. Figure S1. The pseudo-code for the PSO. Figure S2. The pseudo-code for the GA. Figure S3. Flowchart for the complementary GA. Figure S4. Flowchart for the complementary PSO [3,13,28,29]. [file 1471-2156-13-13-S1.DOC]

Supplementary Table 1

Supplementary [Table 1](http://www.plosone.org/article/info%3Adoi%2F10.1371%2Fjournal.pone.0021036#pone-0021036-t001) shows a comparison of the performance *SN*, *SP*, *ACC*, *PC*, and *CC* of different methods from the literature for CpG island prediction. CPSORL provides *SN*, *PC* and *CC* results that are higher than the other methods it was compared to.

Supplementary Table 1. Comparison of different methods for CpG island prediction (CPSO).

| ***Contig.*** | **Methods**  **Performance** | **CpGplot** | **CpG**  **cluster** | **CpGProD** | **CpGIS** | **CpGPSO** | | **CpGCPSO** | |
| --- | --- | --- | --- | --- | --- | --- | --- | --- | --- |
|  |  |  |  |  |  | **without *RL^a^*** | **with *RL*** | **without *RL*** | **with *RL*** |
| **NT_113952.1**  **Length=184355** | SN (%) | 56.43 | 50.46 | 58.07 | 83.98 | 69.22 | 75.58 | 77.43 | **84.88** |
|  | SP (%) | **100.0** | 99.95 | 99.50 | 99.05 | 99.61 | 99.02 | 99.58 | 99.05 |
|  | ACC (%) | 98.09 | 97.78 | 97.69 | 98.39 | 98.28 | 97.99 | **98.61** | 98.43 |
|  | PC (%) | 56.42 | 49.92 | 52.36 | 69.59 | 63.77 | 62.27 | **70.91** | 70.34 |
|  | CC (%) | 74.38 | 69.41 | 68.83 | 81.25 | 77.66 | 75.71 | **82.49** | 81.80 |
| **NT_113955.2**  **Length=281920** | SN (%) | 47.19 | 67.15 | 68.51 | 85.12 | 54.47 | 59.63 | 77.80 | **87.38** |
|  | SP (%) | **100.0** | 99.72 | 99.63 | 99.30 | 99.96 | 99.88 | 99.50 | 99.61 |
|  | ACC (%) | 98.08 | 98.54 | 98.50 | 98.79 | 98.31 | 98.42 | 98.71 | **99.16** |
|  | PC (%) | 47.14 | 62.47 | 62.35 | 71.78 | 53.87 | 57.74 | 68.67 | **79.08** |
|  | CC (%) | 67.94 | 77.03 | 76.65 | 82.96 | 72.41 | 74.51 | 80.85 | **87.89** |
| **NT_113958.2**  **Length=209483** | SN (%) | 51.29 | 27.16 | 46.41 | 82.13 | 79.27 | 81.65 | 81.08 | **84.11** |
|  | SP (%) | **99.99** | 99.94 | 98.93 | 98.26 | 98.13 | 97.90 | 98.17 | 98.34 |
|  | ACC (%) | 96.90 | 95.32 | 95.60 | 97.24 | 96.93 | 96.87 | 97.08 | **97.43** |
|  | PC (%) | 51.24 | 26.92 | 40.10 | 65.36 | 62.10 | 62.33 | 63.80 | **67.51** |
|  | CC (%) | 70.38 | 49.96 | 56.80 | 77.63 | 75.03 | 75.28 | 76.41 | **79.31** |
| **NT_113953.1**  **Length=131056** | SN (%) | 22.80 | 57.32 | 29.79 | 74.05 | 60.20 | 64.80 | 70.53 | **75.65** |
|  | SP (%) | **100.0** | 99.74 | 99.56 | 98.83 | 99.27 | 99.23 | 99.22 | 99.13 |
|  | ACC (%) | 97.76 | **98.51** | 97.53 | 98.11 | 98.13 | 98.23 | 98.38 | 98.45 |
|  | PC (%) | 22.80 | 52.74 | 25.96 | 53.23 | 48.39 | 51.59 | 55.91 | **58.57** |
|  | CC (%) | 47.21 | 69.89 | 43.61 | 68.64 | 64.50 | 67.25 | 70.90 | **73.10** |
| **NT_113954.1**  **Length=129889** | SN (%) | 31.24 | 29.86 | 52.01 | 76.31 | 56.92 | 63.58 | 70.54 | **77.68** |
|  | SP (%) | **100.0** | 99.46 | 98.72 | 97.62 | 98.40 | 98.13 | 98.34 | 98.23 |
|  | ACC (%) | 97.47 | 96.90 | 97.00 | 96.83 | 96.87 | 96.86 | 97.32 | **97.48** |
|  | PC (%) | 31.24 | 26.19 | 38.94 | 47.05 | 40.12 | 42.74 | 49.22 | **53.15** |
|  | CC (%) | 55.17 | 43.81 | 54.68 | 63.29 | 55.65 | 58.36 | 64.72 | **68.53** |
| **NT_028395.3**  **Length=647850** | SN (%) | 27.11 | 44.89 | 54.18 | 76.68 | 68.97 | 72.79 | 72.52 | **77.02** |
|  | SP (%) | **100.0** | 99.47 | 99.45 | 98.93 | 99.27 | 98.99 | 99.18 | 98.90 |
|  | ACC (%) | 97.98 | 97.53 | 98.19 | 98.14 | 98.19 | 98.06 | **98.24** | 98.12 |
|  | PC (%) | 27.10 | 39.26 | 45.36 | 59.36 | 57.49 | 57.17 | **59.36** | 59.25 |
|  | CC (%) | 51.51 | 57.21 | 62.26 | 73.57 | 72.21 | 71.75 | **73.61** | 73.48 |

**Legends:** RL: Reinforcement Learning. SN: Sensitivity. SP: Specificity. ACC: Accuracy. PC: Performance coefficient. CC: Correlation coefficient. Values in bold type represent the best results.

Supplementary Table 2

We compared our method to five other methods reported in the literature, namely CpGplot, CpGProD, CpGcluster, CpGIS and CpGGA. As shown in Supplementary Table 2, the *ACC* of the proposed method was the highest one measured on the NT_113952.1, NT_113955.2, NT_113958.2, NT_113953.1, NT_113954.1 and NT_028395.3 data sets. The proposed method also showed the best prediction performance for *SN*, *PC* and *CC* on all target sequences. Overall, the proposed method obtained better prediction results for CpG islands than the other methods it was compared to.

Supplementary Table 2. Comparison of different methods for CpG island prediction (CpGGA)

| ***Contig.*** | **Methods**  **Performance** | **CpGplot** | **CpG**  **cluster** | **CpGProD** | **CpGIS** | **CpGGA** | | **CpGCGA** | |
| --- | --- | --- | --- | --- | --- | --- | --- | --- | --- |
|  |  |  |  |  |  | **without *RL*** | **with *RL*** | **without *RL*** | **with *RL*** |
| **NT_113952.1**  **Length=184355** | SN (%) | 56.43 | 50.46 | 58.07 | 83.98 | 87.09 | **90.44** | 81.95 | 88.48 |
|  | SP (%) | **100.0** | 99.95 | 99.50 | 99.05 | 98.77 | 98.73 | 99.08 | 98.85 |
|  | ACC (%) | 98.09 | 97.78 | 97.69 | **98.39** | 98.26 | 98.36 | 98.33 | **98.39** |
|  | PC (%) | 56.42 | 49.92 | 52.36 | 69.59 | 68.62 | **70.78** | 68.30 | 70.68 |
|  | CC (%) | 74.38 | 69.41 | 68.83 | 81.25 | 80.67 | **82.35** | 80.29 | 82.16 |
| **NT_113955.2**  **Length=281920** | SN (%) | 47.19 | 67.15 | 68.51 | 85.12 | 81.19 | 83.85 | 83.36 | **88.20** |
|  | SP (%) | **100.0** | 99.72 | 99.63 | 99.30 | 99.86 | 99.78 | 99.89 | 99.79 |
|  | ACC (%) | 98.08 | 98.54 | 98.50 | 98.79 | 99.19 | 99.20 | 99.29 | **99.37** |
|  | PC (%) | 47.14 | 62.47 | 62.35 | 71.78 | 78.34 | 79.22 | 80.89 | **83.52** |
|  | CC (%) | 67.94 | 77.03 | 76.65 | 82.96 | 87.76 | 88.13 | 89.32 | **90.74** |
| **NT_113958.2**  **Length=209483** | SN (%) | 51.29 | 27.16 | 46.41 | 82.13 | 79.79 | **86.43** | 79.82 | 85.25 |
|  | SP (%) | **99.99** | 99.94 | 98.93 | 98.26 | 98.12 | 97.93 | 98.12 | 97.95 |
|  | ACC (%) | 96.90 | 95.32 | 95.60 | **97.24** | 96.96 | 97.20 | 96.96 | 97.15 |
|  | PC (%) | 51.24 | 26.92 | 40.10 | 65.36 | 62.49 | **66.24** | 62.51 | 65.47 |
|  | CC (%) | 70.38 | 49.96 | 56.80 | 77.63 | 75.35 | **78.48** | 75.36 | 77.84 |
| **NT_113953.1**  **Length=131056** | SN (%) | 22.80 | 57.32 | 29.79 | **74.05** | 66.46 | 70.55 | 66.38 | 73.31 |
|  | SP (%) | **100.0** | 99.74 | 99.56 | 98.83 | 99.13 | 99.08 | 99.27 | 99.19 |
|  | ACC (%) | 97.76 | **98.51** | 97.53 | 98.11 | 98.18 | 98.25 | 98.32 | 98.44 |
|  | PC (%) | 22.80 | 52.74 | 25.96 | 53.23 | 51.48 | 53.92 | 53.40 | **57.74** |
|  | CC (%) | 47.21 | 69.89 | 43.61 | 68.64 | 67.05 | 69.17 | 68.85 | **72.40** |
| **NT_113954.1**  **Length=129889** | SN (%) | 31.24 | 29.86 | 52.01 | 76.31 | 75.99 | **81.57** | 72.71 | 74.70 |
|  | SP (%) | **100.0** | 99.46 | 98.72 | 97.62 | 98.25 | 98.19 | 97.78 | 97.74 |
|  | ACC (%) | 97.47 | 96.90 | 97.00 | 96.83 | 97.43 | **97.58** | 96.86 | 96.89 |
|  | PC (%) | 31.24 | 26.19 | 38.94 | 47.05 | 52.14 | **55.38** | 46.03 | 46.95 |
|  | CC (%) | 55.17 | 43.81 | 54.68 | 63.29 | 67.57 | **70.65** | 62.03 | 63.03 |
| **NT_028395.3**  **Length=647850** | SN (%) | 27.11 | 44.89 | 54.18 | 76.68 | 76.46 | **80.13** | 72.40 | 77.43 |
|  | SP (%) | **100.0** | 99.47 | 99.45 | 98.93 | 99.06 | 98.97 | 99.13 | 99.01 |
|  | ACC (%) | 97.98 | 97.53 | 98.19 | 98.14 | 98.25 | **98.30** | 98.19 | 98.25 |
|  | PC (%) | 27.10 | 39.26 | 45.36 | 59.36 | 60.87 | **62.55** | 58.62 | 61.04 |
|  | CC (%) | 51.51 | 57.21 | 62.26 | 73.57 | 74.77 | **76.15** | 72.99 | 74.92 |
| **Legends:** RL: Reinforcement Learning. SN: Sensitivity. SP: Specificity. ACC: Accuracy. PC: Performance coefficient. CC: Correlation coefficient. Values in bold type represent the best results. | | | | | | | | | |

Supplementary Table 3. Comparison of different methods on the number of CpG islands identified in the entire human genomes.

| Methods | CpGcluster | CpGIS | Bock et al. | CPSORL |
| --- | --- | --- | --- | --- |
| Genome length | 2.86E+09 | | | |
| Number of predicted islands | 198,702 | 37,729 | 109,510 | 208,536 |
| Average of island length | 273 | 1,090 | 465 | 572 |
| GC content | 63.78 | 60.61 | 56.20 | 53.90 |
| CpG island O/E ratio | 0.855 | 0.717 | 0.676 | 0.649 |

**Supplementary Algorithm CGA**

GA is a stochastic search algorithm modeled after the process of natural selection that underlies biological evolution. The pseudo-code of GA for the prediction of CpG island is shown in supplementary Figure 2. The standard GA procedure applies the following genetic operators: chromosome encoding and initialization, selection, crossover and mutation, which is the process by which a whole generation of new offspring is computed. By applying genetic operators on strings in the mating pool, a new population of strings is formed in the next generation. If the fitness of a chromosome in CGA does not change after five iterations, the chromosome position is changed by the complementary operation. The implementation of the genetic operators is repeated in each subsequent generation until a termination condition is reached. The flowchart of CGA is shown in supplementary Figure 3, and a detailed description of CGA for CpG island prediction is shown below:

***Complementary Genetic Algorithm***

A genetic algorithm was first proposed in the 70s [1], and researchers have since been investigating various methods for enhancing this algorithm. A GA is a stochastic search algorithm modeled after the process of natural selection which underlies biological evolution. The basic concept behind a GA is to design and simulate evolutionary processes in natural systems, specifically those that follow this principle of survival of the fittest first laid down by Charles Darwin. As such, they represent an intelligent exploitation of a random search within a defined search space to solve a problem. The CpGCGA consists of several major steps, namely the encoding of the chromosome and its initialization, the fitness evaluation, the selection, crossover and mutation operators, a replacement process and a complementary operation. The length of the CpG islands influences the prediction performance. Longer CpG islands are preferable to shorter ones. For this reason we used reinforcement learning (RL) to extend shorter CpG islands and combine them with neighbouring CpG islands.

## *A. Chromosome Encoding and Initialization*

CpG islands are predicted by a two-dimensional string. The massive bulk of the DNA sequence is separated into many relatively small sections (blocks). The individual prediction accuracy *Pv* of a CpG island can be represented by *Pv = (Fs, F_l_)*, where *Fs* is the start site of an island fragment and *F_l_* denotes the randomly generated length of a CpG island. *Fs* is randomly generated and *F_l_* denotes the randomly generated length of a CpG island between 200 and 2000 bp.

## *B. Fitness Evaluation*

We based the fitness evaluation of a GA’s chromosomes on the criteria proposed by GGF [2]. The length of CpG islands generally varies from 200 ~ 2000 bp [3]. If the length of CpG islands is directly added into the fitness function, the resulting value can not directly be used for a comparison to the originally proposed criteria. This necessitates a length reduction. In order to reduce the length, we adopt a normalization function for each length. This function reduces the length value of the CpG islands and leads to values within a small range (i.e., a CpG island length of 200-2000 bp; after application of the length reduction function, the value was in the range of 0 to 1). The length value function is shown in Eq. 1.

As stated previously, CpG islands are a short string of DNA, in which the frequency of sequences containing the nucleotides C and G is higher than in other regions of the DNA molecule. Hence, it may be assumed that CpG islands with a higher GC content and CpGs *O/E* ratio value may be more significant. The GC content and CpGs *O/E* ratio functions are given by Eq. 2 and Eq. 3. The fitness value is given by the fitness function in Eq. 4.

| $CpG_{length\_value}=\left\{\begin{matrix} \text{ }\frac{CpG\_length-\text{Len(min)}}{\text{Len(max)-Len(min)}}\text{ , if CpG\_length}>=\text{200 and CpG\_length}<=\text{2000 } \\ \text{ }0,\text{ otherwise} \end{matrix}\right.$(1) | [1] |
| --- | --- |
| $GC=\frac{\#GC}{\#A\_T\_C\_G}$ (2) | [2] |
| (3) | [3] |
| (4) | [4] |

## *C. Selection, Crossover and Mutation*

Viable modes of selection for individuals in a GA include tournament selection and roulette wheel selection. The selection operation must ensure that selected CpG islands have a high fitness value. We adopted a rank-based tournament selection scheme in the study. Two solutions from the population are selected, their fitness values are compared and recorded, and then the best solutions are ranked. We used the standard crossover and mutation operation from the tournament selection to select two parents, P_1_ and P_2_. Two offsprings, S_1_ and S_2_, were produced by the exchange of information between the two parents P_1_ and P_2_. However, assuming that fragments of the CpG islands (*F_s_ + F_l_*) are bigger than the block (*size* = 3000), a mechanism for adjusting the length has to be implemented.

## *D. Replacement Operation*

In a GA, the crossover operation generates offsprings of two parents, and the mutation operation slightly perturbs these offspring. If an offspring is superior to both parents, it replaces the most similar parent; if an offspring’s fitness lies between the fitness of the two parents, it replaces the inferior parent; otherwise, the most inferior GA’s chromosome in the population is replaced.

## *E. Complementary Operation*

Each chromosome produces new offsprings based on crossover and mutation operations. The operation adjusts the fitness of a chromosome search position. If the fitness of a chromosome does not change after five iterations, it is considered stuck in a local optimum. This behavior may make it impossible to predict new CpG islands.

The CpGCGA proposed in this study prevents the entrapment of particles in a local optimum by introducing a complementary operation. Under such circumstances, we allow this chromosome to change its position. The current *F_s_* and *F_l_* are changed by a complementary operation, i.e., the chromosome searches for the next block. Each *F_s_* and *F_l_* are changed based on the following Eq. 5 and Eq. 6.

| *Complementary Rule:* |  |
| --- | --- |
| $New\_F_{s}=(block_{Max}+block_{Min})-(F_{s\_Current})+\frac{blocksize}{2}$ | (5) |
| $New\_F_{l}=(Len_{Max}+Len_{Min})-(F_{l\_Current})$ | (6) |

In these equations, *block_Max_* and *block_Min_* are the maximized and minimized base pairs in the block, *Len_Max_* is set to 2500 (i.e., *block size* – length criteria) and *Len_Min_* is based on the length criteria. When allowing the GA’s worst chromosome to search for other blocks, *Fs_Current* has to be increased by$blocksize/2$, so that CpG islands can be searched for in the new blocks (i.e., global search takes place). On the other hand, the GA’s superior chromosomes conduct their search around the current position (local search).

## *F. Reinforcement Learning*

Reinforcement learning (RL) is an approach to intelligence control [4]. RL combines dynamic programming and supervised learning to yield powerful machine-learning systems. RL uses internal predictive models to improve the learning rate and tries various output states to search for the best result. The results are evaluated repeatedly until a predefined termination criterion is reached. A RL system can be viewed as a machine whose target is to maximize the positive (correct) and minimize the negative (incorrect) results. CpG islands that conform to the GGF criteria [1] are predicted by CpGCGA. However, the length of a predicted CpG islands sequence is shorter than experimentally verified CpG island sequences. We thus used RL to extend the length of the predicted CpG islands. If the length between adjacent CpG islands is shorter than 200 bp, the two CpG islands are combined. After that, all predicted CpG islands are extended until the defined criterion is not satisfied anymore.

As stated above, the length of some predicted CpG islands is often shorter than known CpG islands; some CpG islands are also located within close proximity of each other. This may greatly influence the sensitivity (*SN*). To overcome this problem, some methods based on the sliding window technique were developed. However, with this technique some assumed target sequences become so long that the resulting computation time is unacceptable. For this reason, we used RL to extend the length of the CpG islands in this study. If two CpG islands are within close proximity of each other then the extension operation is performed. The sign of the scalar reinforcement at the terminal state indicates whether the terminal state is a goal state (a reward) or a state that should be avoided (a penalty).

**References**

1. Holland JH: **Adaptation in Natural and Artificial Systems**. In*.*: University f Michigan Press; 1975.
2. Gardiner-Garden M, Frommer M: **CpG Islands in vertebrate genomes* 1**. *Journal of molecular biology* 1987, **196**(2):261-282.
3. Fang F, Fan S, Zhang X, Zhang MQ: **Predicting methylation status of CpG islands in the human brain**. *Bioinformatics* 2006, 22:2204-9.
4. Whitehead S, Sutton R, Ballard D: **Advances in reinforcement learning and their implications for intelligent control**, In: *Proceedings of the 5th IEEE Int. Symposium on Intelligent Control* 1990, 1289-1297.

### Supplementary Figure 1.

The pseudo-code for the PSO is shown below.

***P***seudo-code for PSO

| Pseudo-code for PSO |
| --- |
| 1. **Begin**  2. Randomly initialize particles swarm  3. **while**(the stopping criterion is not met)  4. Evaluate fitness of particles  5. **For** n = 1 to number of particles  6. Find *pbest*  7. Find *gbest*  8. **For** d=1 to number of dimension of particle  9. update the position of particles by Eq. (1)-(2)  10. **Next** d  11. **Next** n  12. update the inertia weight value by Eq.(3)  13. **Next** generation until stopping criterion  14. **End** |

(1)

$x_{id}^{new} =x_{id}^{old} +v_{id}^{new}$ (2)

$w=(w_{\max-}w_{\min})\times\frac{move_{\max-}move_{i}}{move_{\max}}+w_{\min}$ (3)

### Supplementary Figure 2.

A pseudo-code for the GA is shown below.

***Pseudo-code for GA***

| Pseudo-code for GA |
| --- |
| 1. **Begin** 2. Set window size 3. Randomly generate initial population 4. Calculate the fitness of each chromosome 5. **For** *i* = 1 to number of generations 6. Select the two parents *i_a_* and *i_b_* via tournament selection 7. Generate offspring*_i_* = crossover (*i_a_* and *i_b_*) 8. Randomly generate value of *r* 9. **If** ( *r* > mutation ratio) 10. mutation (offspring*_i_*) 11. Replace the worst parents and chromosome. 12. **Next** *i* 13. **End** |

**Supplementary Figure 3.**

Flowchart for the complementary GA.

**Supplementary Figure 4.**

Flowchart for the complementary PSO is shown below.

Illustrative example:

To predict CpG islands, each particle is encoded as *P_i_= (Fs, Fe)*, where *Fs* and *Fe* represent the start and end positions of a CpG island, respectively. In the example below, the population size is 3, and *C*_1_ and *C*_2_ are set to 2. The sequence length is 10,000 bp, i.e., we limit *Fe* to 10,000.


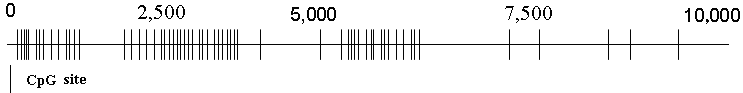


Step 1: particle initialization

*P_1_ = (2500, 4000)*

*P_2_ = (5100, 6200)*

*P_3_ = (8000, 10000)*

Step 2: Evaluate fitness of *P­_i_* by using Eq. (1-4)

| *CpG_length(max)_=2000, CpG_length(min)_=200* | (1) |
| --- | --- |
| $GC(P_{i})=\frac{\#C+\#G}{\#A+\#T+\#C+\#G}$ | (2) |
| $\text{Obs}_{\text{CpG}}\text{/Exp}_{\text{CpG}}\text{ }(P_{i})=\frac{\frac{\#CpG}{CpG_{length}}}{\frac{\#C}{CpG_{length}}\times\frac{\#G}{CpG_{length}}}$ | (3) |
| $Fitness(P_{i})=GC(P_{i})+Obs_{CpG}/Exp_{CpG}(P_{i})+CpG_{length} (P_{i})$ | (4) |

#A: number of A (Adenine), #T: number of T (Thymine), #C: number of C (Cytosine) and #G: number of G (Guanine) nucleotides in the CpG islands represented by particle *P­_i_*. #CpG: number of CpG islands. *CpG_length_*: length of CpG island.

Step 3: Evaluate fitness of each particle *P_i_*


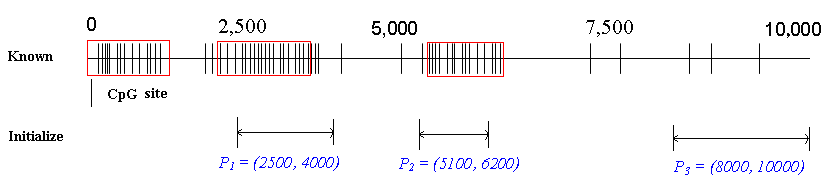


*P_1_ = (2500, 4000),* the length of CpG island (*CpG_length_*) is 1,500 (4000-2500) bp. If the number of C (*#C*) is 750, the number of G (*#G*) is 700 and the number of CpG (*#CpG*) is 280. The fitness (*P_1_*) is thus calculated as:

$$Fitness(P_{1})=\frac{750+700}{1500}+\frac{\frac{280}{1500}}{\frac{750}{1500}*\frac{700}{1500}}+\frac{(4000-2500)-200}{2000-200}=0.96+0.8+0.72=2.48$$

*P_2_ = (5100, 6200),* the length of CpG island (*CpG_length_*) is 1,100 (6200-5100) bp. If the number of C (*#C*) is 500, the number of G (*#G*) is 450 and the number of CpG (*#CpG*) is 200. The fitness (*P_2_*) is thus calculated as:

$$Fitness(P_{2})=\frac{500+450}{1100}+\frac{\frac{200}{1100}}{\frac{500}{1100}*\frac{450}{1100}}+\frac{(6200-5100)-200}{2000-200}=0.86+0.98+0.5=2.34$$

*P_3_ = (8000, 10000),* the length of CpG island (*CpG_length_*) is 2,000 (10000-8000) bp. If the number of C (*#C*) is 100, the number of G (*#G*) is 150 and the number of CpG (*#CpG*) is 10. The fitness (*P_3_*) is thus calculated as:

$$Fitness(P_{3})=\frac{100+150}{2000}+\frac{\frac{10}{2000}}{\frac{100}{2000}*\frac{150}{2000}}+\frac{(10000-8000)-200}{2000-200}=0.125+1.333+0.5=1.96$$

The fitness of *pbest_1_* is 2.48, the fitness of *pbest_2_* is 2.34 and the fitness of *pbest_3_* is 1.96. Since the fitness value of *P_1_* is the maximum value, *pbest* is now the *gbest*: *gbest =pbest_1._*

*
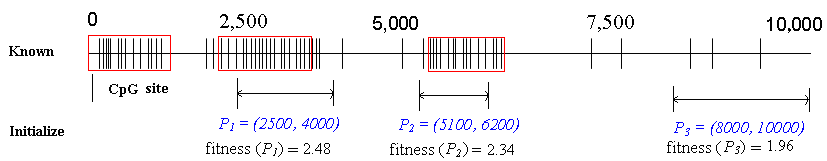
*

Step 4: If *gbest* has not improved for five iterations then half of the population is randomly selected and replaced by complementary particles to increase the search space.

Suppose that *P_3_*= (8000, 10000) is selected; we use Eq. (5) to generate the complementary particle.

$x_{id}^{complement}=(X_{\max}+X_{\min})-x_{id}^{selected}$ (5)

where$x_{id}^{selected}$is the position of the randomly selected particle, and $x_{id}^{complement}$is the position of the respective complementary particle.$X_{\max}$and$X_{\min}$denote the maximum (10000,10000) and minimum (0,0) limit of the solution space, respectively.

$$\text{P}_{3}^{complement}=[(10000,10000)+(0,0)]-(8000,10000)=(18000,\text{ 20000})$$

where 18000 represents the start site, and 20000 represents the end site, but the result surpass the limit. Hence, we random the *P_3_,* if the random result are *P_3_= (0, 2000).*

Evaluation of all particles

*P_3_= (0, 2000)**,* the length of the CpG island (*CpG_length_*) is 2,000 (2000-0) bp. If the number of C (*#C*) is 680, the number of G (*#G*) is 610 and the number of CpG (*#CpG*) is 300. The fitness (*P_3_*) is thus calculated as:

$$Fitness(P_{3})=\frac{680+610}{2000}+\frac{\frac{300}{2000}}{\frac{680}{2000}*\frac{610}{2000}}+\frac{(2000-0)-200}{2000-200}=0.64+1.44+1=3.08$$

The fitness of *pbest_1_* is 2.48, the fitness of *pbest_2_* is 2.34 and the fitness of *pbest_3_* is 3.08. Since the fitness value of *P_3_* is the highest, *pbest_3_* is now the *gbest*: *gbest = pbest_3._*

Step 5: Update velocity (*V_i_* ) and position (*X_i_*)

At each generation, the position and velocity of every particle is updated according to its own *pbest* and *gbest* by Eq. (6) and Eq. (7).

|  | (6) |
| --- | --- |
| $x_{id}^{new} =x_{id}^{old} +v_{id}^{new}$ | (7) |

where *r_1_* and *r_2_* are random numbers between (0, 1).

Update of *V_1_* and *X_1_*

$v_{1}^{old}$

If *w* is 1, is (1, 1), *r_1_* is 0.01, *r_2_* is 0.02, and both *C_1_* and *C_2_* are 2.

Update *V_2_* and *X_2_*

$v_{2}^{old}$

If *w* is 1, is (1, 1), *r_1_* is 0.02, *r_2_* is 0.01, *and C_1_* and *C_2_* are 2, respectively.

Update of *V_3_* and *X_3_*

$v_{3}^{old}$

If the *w* is 1, is (1, 1), *r_1_* is 0.1, *r_2_* is 0.2, and *C_1_* and *C_2_* are 2, respectively.

Step 6: If the stopping criterion is satisfied then the particle results are output.

*P_1_= (2401, 3921),* the length of CpG island (*CpG_length_*) is 1,520 (3921-2401) bp. If the number of C (*#C*) is 420, the number of G (*#G*) is 430 and the number of CpG (*#CpG*) is 200. The fitness (*P_1_*) is thus calculated as:

$$Fitness(P_{1})=\frac{420+430}{1520}+\frac{\frac{200}{1520}}{\frac{420}{1520}*\frac{430}{1520}}+\frac{(3921-2401)-200}{2000-200}=0.56+1.69+0.73=2.98$$

The updated Fitness (*P_1_*) is 2.98, which is better than the original *pbest* fitness. Hence, *pbest*_1_ = (3921, 2401), and its fitness is 2.98.

*P_2_ = (4999, 6077)* the length of CpG island (*CpG_length_*) is 1,078 (6077-4999) bp. If the number of C (*#C*) is 510, the number of G (*#G*) is 500 and the number of CpG (*#G*) is 180. The fitness (*P_2_*) is thus calculated as:

$$Fitness(P_{2})=\frac{510+500}{1078}+\frac{\frac{180}{1078}}{\frac{510}{1078}*\frac{500}{1078}}+\frac{(6077-4999)-200}{2000-200}=0.94+0.76+0.49=2.19$$

The updated fitness (*P_2_*) is 2.19. Since *pbest_2_* is 2.48, *pbest_2_* again remains unchanged.

*P_3_= (1, 2001),* the length of the CpG island (*CpG_length_*) is 2,000 (2001-1) bp. If the number of C (*#C*) is 680, the number of G (*#G*) is 610 and the number of CpG (*#CpG*) is 300. The fitness (*P_3_*) is thus calculated as:

$$Fitness(P_{3})=\frac{680+610}{2000}+\frac{\frac{300}{2000}}{\frac{680}{2000}*\frac{610}{2000}}+\frac{(2001-1)-200}{2000-200}=0.64+1.44+1=3.08$$

The updated fitness (*P_3_*) is 3.08, Since *pbest3* is 2.48, *pbest_3_* again remains unchanged.

The final result is

*P_1_= (2401,3921),* Fitness (*P_1_*) =2.98

*P_2_= (4999,6077),* Fitness (*P_2_*) =2.19

*P_3_= (1,2001),* Fitness (*P_3_*) =3.08

Step 7: RL system


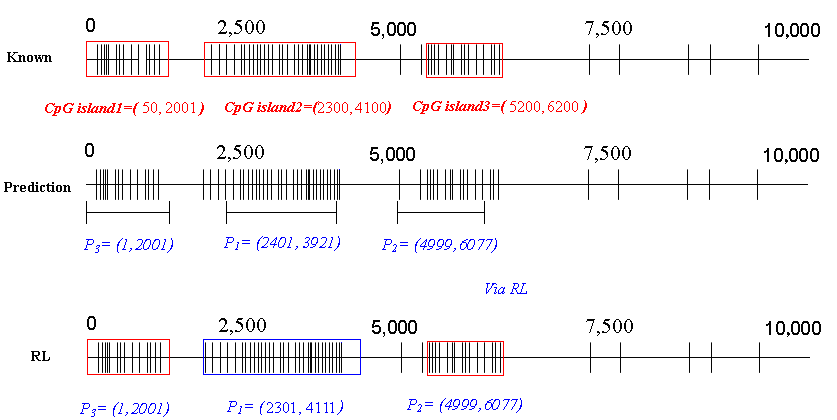


The RL system is applied to extend each CpG island while the prediction still conforms to GGF criteria (not considering the CpG island length). The RL system is then applied to the results of the CPSO algorithm. For example, the CpG island encoded by *P_1_* is extended from (*2401, 3921)* to (*2301, 4111*) by altering the start and end positions.

Example:

The figure above shows that for the known CpG island1 located at 2300 bp~4100 bp, a predicted CpG island is located between 2401 and 3921 before RL is applied (2401*,* 3921*)*. After the extension, the length of the CpG island (*CpG_length_*) is 1810 (2300, 4110) bp and the prediction still remains conform to the GGF criteria (Length≧200, GC content≧0.5 and observed/expected (O/E) ratio≧0.6).

For the original *P_1_= (2401, 3921),* the length of CpG island (*CpG_length_*) is 1,520 (3921-2401) bp. If the number of C (*#C*) is 420, the number of G (*#G*) is 430 and the number of CpG (*#CpG*) is 200. The fitness (*P_1_*) is thus calculated as:

$$Fitness(P_{1})=\frac{420+430}{1520}+\frac{\frac{200}{1520}}{\frac{420}{1520}*\frac{430}{1520}}+\frac{(3921-2401)-200}{2000-200}=0.56+1.69+0.73=2.98$$

In the next steps, the RL system is used to extend the CpG island by 10 bp (left shift the start position and right shift the end position by 5bp). *P_1_* is thus extended from (*2401, 3921)* to (2396*, 3926*). If the number of C (*#C*) is 420, the number of G (*#G*) is 430 and the number of CpG (*#CpG*) is 200.The fitness (*P_1_*) is calculated as:

$$Fitness(P_{1})=\frac{420+430}{1530}+\frac{\frac{200}{1530}}{\frac{420}{1530}*\frac{430}{1530}}+\frac{(3926-2396)-200}{2000-200}=0.56+1.69+0.73=2.98$$

The GC content or O/E ratio of each extended CpG island is repeatedly calculated until the GGF criteria are not conformed to anymore (not include CpG island length here). *P_1_* is extended from (2396*, 3926)* to (2301*, 4021*). If the number of C (*#C*) is 420, the number of G (*#G*) is 430 and the number of CpG (*#CpG*) is 200. The fitness (*P_1_*) is calculated as:

$$Fitness(P_{1})=\frac{430+430}{1720}+\frac{\frac{200}{1720}}{\frac{420}{1720}*\frac{430}{1720}}+\frac{(4221-2301)-200}{2000-200}=0.5+1.86+0.84=3.2$$

In the next step both ends of the predicted island are simultaneously extended. If the number of C (*#C*) is 420, the number of G (*#G*) is 430 and the number of CpG (*#CpG*) is 200. *P_1_* is extended from (*2301, 4021)* to (*2296, 4026*). The fitness (*P_1_*) is calculated as:

$$Fitness(P_{1})=\frac{420+430}{1730}+\frac{\frac{200}{1730}}{\frac{420}{1730}*\frac{430}{1730}}+\frac{(4026-2296)-200}{2000-200}=0.49+1.91+0.85=3.25$$

Since the island has a GC content < 0.5, the previous step needs to be rolled back and the start position left-shifted by 10 bp. If the number of C (*#C*) is 420, the number of G (*#G*) is 430 and the number of CpG (*#CpG*) is 200. *P_1_* is extended from *(2301, 4021)* to (*2296, 4021*). The fitness (*P_1_*) is calculated as:

$$Fitness(P_{1})=\frac{420+430}{1730}+\frac{\frac{200}{1730}}{\frac{420}{1730}*\frac{430}{1730}}+\frac{(4026-2296)-200}{2000-200}=0.49+1.91+0.85=3.25$$

Given that the island has a GC content < 0.5, we again need to roll back the previous step and right shift the end position by 10 bp. If the number of C (*#C*) is 430, the number of G (*#G*) is 430 and the number of CpG (*#CpG*) is 200. *P_1_* is extended from *(2301, 4021)* to (2*301, 4031*). The fitness (*P_1_*) is calculated as:

$$Fitness(P_{1})=\frac{430+430}{1730}+\frac{\frac{200}{1730}}{\frac{430}{1730}*\frac{430}{1730}}+\frac{(4031-2301)-200}{2000-200}=0.5+1.87+0.85=3.22$$

Since the extended island has a GC content ≧ 0.5 and conforms to GGF criteria, RL continues to left shift the start position; *P_1_* is extended from *(2301, 4031)* to *(2301, 4111)*. If the number of C (*#C*) is 450, the number of G (*#G*) is 450 and the number of CpG (*#CpG*) is 200.The fitness (*P_1_*) is calculated as below:

$$Fitness(P_{1})=\frac{450+450}{1810}+\frac{\frac{200}{1810}}{\frac{450}{1810}*\frac{450}{1810}}+\frac{(4111-2301)-200}{2000-200}=0.49+1.78+0.89=3.17$$

Given that the GC content < 0.5, the previous step is undone and RL terminated since the stopping criterion has been reached. Finally, *P1* is extended from *(2301, 4031)* to *(2301, 4111)*.

*P_1_= (2301, 4111)*

*P_2_= (4999, 6077)*

*P_3_= (1, 2001)*

This result indicates that the CpG islands are located between 1~ 2001 bp, 2301~4111 bp and 4999~6077 bp, respectively.
